# Selective perturbation of cognitive conflict in the human brain–A combined fMRI and rTMS study

**DOI:** 10.1038/srep38700

**Published:** 2016-12-13

**Authors:** Claudia Peschke, Raphael Köster, Margarethe Korsch, Sascha Frühholz, Christiane M. Thiel, Manfred Herrmann, Claus C. Hilgetag

**Affiliations:** 1 Department of Computational Neuroscience, University Medical Center Hamburg-Eppendorf, Hamburg, Germany; 2 School of Humanities and Social Sciences, Jacobs University Bremen, Bremen, Germany; 3 Center for Advanced Imaging, Department of Neuropsychology and Behavioral Neurobiology, Bremen University, Bremen, Germany; 4 Institute of Cognitive Neuroscience, University College London, London, United Kingdom; 5 Swiss Center for Affective Sciences, University of Geneva, Geneva, Switzerland; 6 Biological Psychology Lab, Department of Psychology, European Medical School, Carl von Ossietzky University Oldenburg, Oldenburg, Germany; 7 Department of Health Sciences, Boston University, Boston, MA, USA

## Abstract

We investigated if single and double conflicts are processed separately in different brain regions and if they are differentially vulnerable to TMS perturbation. Fifteen human volunteers performed a single (Flanker or Simon) conflict task or a double (Flanker and Simon) conflict task in a combined functional Magnetic Resonance Imaging (fMRI) and Transcranial Magnetic Stimulation (TMS) study. The fMRI approach aimed at localizing brain regions involved in interference resolution induced by single Flanker (stimulus-stimulus, S-S) and Simon (stimulus-response, S-R) conflicts as well as regions involved in the double conflict condition. The data revealed a distinct activation in the right intraparietal sulcus (IPS) for Flanker interference and in the right middle frontal gyrus (MFG) for the double interference condition. The causal functional role of these brain regions was then examined in the same volunteers by using offline TMS over right IPS and right MFG. TMS perturbation of the right IPS increased the Flanker effect, but had no effect in the Simon or double conflict condition. In contrast, perturbation of the right MFG had no effect on any of the conflict types. These findings suggest a causal role of the right IPS in the processing of the single conflict of Flanker (stimulus-stimulus) interference.

Cognitive control of parallel, interfering signaling streams is an essential brain function that influences behavior through modulating multiple stages of perception, cognition and action, by extracting relevant information and inhibiting irrelevant aspects in the face of competing input information. Several parieto-frontal brain regions guide the prevention of interference by irrelevant stimulus information that competes with the relevant target information for visual attention and behavioral responses. Here we used transcranial magnetic stimulation (TMS) based on functional magnetic resonance imaging (fMRI) group data to examine the causal role of these brain regions.

The ability to resolve conflicting information is frequently investigated with experimental paradigms such as the Flanker task or the Simon task. In a typical Flanker task^1^,^2^ participants are asked to respond to a central stimulus (e.g., a letter or arrow) by pressing one out of two possible response buttons while ignoring irrelevant flanker stimuli that are presented simultaneously to the left and right of the target stimulus. The flankers can either be associated with the same response as the target (congruent condition) or the alternative response (incongruent condition). Typically, participants’ performance is worse in the incongruent condition where the task-irrelevant information of the flanking stimuli is mapped to a different response than in the task-relevant information of the target stimulus. In contrast, in a classical Simon paradigm, the target stimulus is usually confounded with irrelevant spatial information, for instance, through the lateralized presentation of the target. Here, the stimulus can either be shown on the same side as the expected response (congruent condition) or on the opposite side (incongruent condition). As in the Flanker task, participants show a worse performance in the incongruent condition. There is an ongoing debate about whether these distinct sorts of conflicts occur independently or rather interact during information processing. Kornblum *et al*.^3^ introduced the “Dimensional Overlap” (DO) model which suggests that the Flanker and Simon conflict represent distinct conflict types that occur in different stages of information processing. The Flanker conflict is defined by interfering stimulus characteristics of the target stimulus and the irrelevant stimuli (stimulus-stimulus (S-S) conflict), and usually is associated with an early stage of stimulus identification. In contrast, the Simon conflict is based on the overlap of the irrelevant stimulus dimension (e.g. left vs. right lateralized stimulus presentation) and the response set (left vs. right reaction/button press; stimulus-response (S-R) conflict) and is supposed to evolve later at the response preparation stage. The DO model was substantiated by findings of additive single conflict effects in combined S-S and S-R tasks^4^,^5^,^6^,^7^. Li *et al*.^8^ demonstrated that also with varying stimulus characteristics, the combination of S-S and S-R conflicts leads to additive effects. Further evidence came from studies that revealed conflict specific trial-sequence-effects^9^,^10^, as well as studies that demonstrated specific effects on different types of conflict resolution in aging^11^,^12^,^13^. However, there are also studies that demonstrated a clear interaction between the Stroop or Flanker paradigm and the Simon task and thus challenge basic assumptions of the DO model^14^,^15^.

In recent years different methodological approaches such as EEG, fMRI and TMS have been used to investigate whether both conflict types also interact or dissociate with regard to their underlying neural correlates. Several EEG studies demonstrated that the temporal characteristics of conflict processing are specific for different conflict types as assumed by the DO model^16^. Further, there exists prior evidence that different types of conflict, as well as the combination of different conflict types rely on distinct neural mechanisms^7^. In general, fMRI studies have shown that conflict-inducing tasks activate a widely distributed bilateral neural network including the posterior parietal cortex (PPC), the dorsolateral prefrontal cortex (DLPFC), the anterior cingulate cortex (ACC) and inferior frontal regions^17^,^18^,^19^. In particular, the parietal cortex in the vicinity of the inferior parietal sulcus (IPS) is assumed to be involved in conflict resolution by orienting attention to the relevant target^17^,^20^,^21^, and by representing possible responses^22^,^23^. The DLPFC and ACC are proposed to monitor conflicting information^17^,^20^,^21^,^24^ and to select among competing responses^18^,^22^,^25^,^26^. Moreover, the inferior frontal gyrus (IFG) in turn is presumed to guide the inhibition of responses to irrelevant information^23^,^27^,^28^. Despite the considerable number of existing studies, it remains difficult to determine whether neural networks underlying conflict processing are specific for different conflict types. A meta analysis by Nee *et al*.^18^ clearly suggests that the processing of different conflict types is linked to distinct neural networks. While interference resolution during Flanker (S-S) conflict processing is mainly associated with an activation of the DLPFC, S-R interference seems to be associated with an increased activation of the posterior parietal cortex, the anterior cingulate and the premotor cortex.

However, the majority of studies focused on only one single type of conflict and, additionally, represent a huge variability regarding the study samples, study design, stimulus material, methodological approaches etc. that also account for different activation patterns across paradigms. There is only a sparse number of studies that investigated these different conflict types within one setting. Egner *et al*.^10^ analyzed sequential trial effects in a combined Stroop and Simon conflict task and reported stimulus-based control mechanisms (Stroop task) to be associated with activation of the superior parietal lobule. In contrast, the activation of response-based control mechanisms (Simon task) elicited increased activity in the premotor cortex. A previous study of our group also indicated distinct brain mechanisms for different types of conflict^7^. We used fMRI and electroencephalography (EEG) to compare the underlying neural networks and the temporal features of a S-S and S-R conflict processing. The fMRI data revealed that the S-S conflict predominantly activated the right inferior frontal cortex, the superior parietal cortex and the dorsal ACC. In contrast, the S-R conflict revealed more sparse activations especially in the anterior medial and superior frontal cortex and in the posterior cingulate cortex. The combined double conflict condition resulted in an activation of the anterior fronto-polar cortex and the superior parietal cortex. This pattern of distinct brain mechanisms was confirmed by the EEG data that suggest a sequential processing of Flanker and Simon conflicts during double conflict trials^7^. Taken together, previous studies indicate that the parietal cortex is involved in the processing of S-S conflicts. However, it remains unclear whether the prefrontal cortex is critical for the processing of different conflicts. Furthermore, the data regarding the S-R conflict seem to be inconsistent. Thus, it remains open whether S-S and S-R conflict processing rely on specific and distinctive neural networks. Another question that cannot be answered with fMRI is whether the neural sites that are activated in various studies play a critical role for the processing of the different conflicts or whether the activation rather represents additional cognitive resources that are not crucial for conflict resolution or even just an epiphenomenon.

To approach these issues, the present study proceeded in two steps. First, fMRI was used to individually localize brain regions involved in interference resolution induced by two different single conflicts as well as the combined double conflict, respectively. Then, TMS was used to investigate these regions’ causal contribution to conflict processing. To implement different types of conflict, we combined Flanker and Simon-like paradigms that were expected to represent S-S and S-R conflict tasks, respectively. In contrast and complementary to imaging approaches which provide correlational relationships, TMS enables the examination of causal relationships between brain activity and behavior, and thus can deliver unique, causal information about the location and dynamics of the functional contribution of brain regions. There is extensive evidence that TMS of frontal and parietal cortical sites interferes with stimulus detection^29^,^30^,^31^, spatial attention^32^,^33^, and feature-based visual search^33^,^34^,^35^,^36^. However, there are only few studies using TMS to examine the effects of attention in conflict tasks (for review see ref. 37). A contribution of the right PPC to spatial attention was observed in a previous study using unilateral flankers^38^. TMS of the right PPC abolished the effect of flankers shown in the contralateral left visual field, while the effect of ipsilateral flankers persisted. This finding might be due to reduced attention to flankers in the left visual field after TMS of the right PPC and, thus, a decreased flanker effect. In a recent study using bilateral flankers, it was also shown that TMS of the same region in PPC interfered with perceptual conflict processing; however, the stimulation also facilitated the processing of response selection conflict^39^. A modulation of S-R conflict resolution by TMS of the parietal cortex was also observed by Schiff *et al*.^40^.

Regarding the frontal cortex, a direct causal role in conflict resolution in a Flanker task was demonstrated for the dorsal medial frontal cortex^41^. Another stimulation study using tDCS^42^ demonstrated that cathodal stimulation of the right DLPFC led to increased reaction times in trials with incongruent Flanker stimuli but did not affect the Simon conflict. TMS studies using conflict tasks predominantly focused on effects at the response selection level when applying TMS over the (pre-)motor cortex^39^,^43^,^44^,^45^.

In summary, fMRI and TMS studies revealed that conflict processing is strongly associated with parietal cortices, the premotor cortex, but also prefrontal areas. However, due to the lack of studies that investigate different conflict types within a single experimental setting, it remains unclear which brain regions contribute causally to particular S-S and S-R conflicts, whether these brain areas are distinctive for the different conflict types and which functional role they play in the processing of conflicting information. Furthermore, it is not known whether some brain regions contribute to specific combinations of conflicts or are predominantly involved because of increased cognitive load in multiple conflict situations.

In the present study, we went beyond a correlational neuroimaging approach by using TMS to perturb different brain regions in order to establish their specific causal functional contributions in conflict resolution. The previously mentioned TMS studies have in common that the stimulation sites were determined *a priori* in a theory-driven way. In the present combined fMRI-TMS study, fMRI was used to empirically detect the brain regions involved in single conflict and double conflict resolution. Two of these regions, in the vicinity of the right intraparietal sulcus (IPS) and the right middle frontal gyrus (MFG) were then selected to examine whether one conflict condition, but not the other ones, could be selectively perturbed when applying TMS to the distinct brain regions. Specifically, we investigated the hypotheses that TMS of the IPS might selectively interfere with the S-S conflict condition, while TMS of MFG might either selectively interfere with the combined S-S and S-R double conflict condition, or might affect the double conflict condition as well as one or more of the single conflict conditions. In the latter case of interference with the double as well as single conflict conditions, one might be able to conclude that MFG generally contributes to the increased cognitive load in the double conflict condition. Our findings, detailed in the remainder of the paper, confirm a causal contribution of IPS to S-S (Flanker) conflict processing, but were unable to establish a causal role of MFG in the processing of cognitive conflict.

## Materials and Methods

### Participants

fMRI data were obtained for a total of 17 subjects (eight females, mean age 24.2 years; SD = 3.1 years; age range 20–31 years). Of these 17 participants, two dropped out during the TMS sessions, so that the analysis of the TMS data was based on 15 participants (six females, mean age 23.9 years; SD = 2.7 years; age range 20–31). All of them were right-handed (based on ref. 46), had normal or corrected to normal vision, no history of neurological or psychiatric disorder and were free of medication. The study was designed and conducted in accordance with the Declaration of Helsinki. All experimental procedures were approved by the German Psychological Society (DGPs) and written informed consent was obtained from the participants at every stage of the experiment. Subjects were paid for their attendance after each session.

### Procedure and experimental paradigm

Each participant took part in four sessions. The aim of the first two sessions was to measure conflict-related neural activity. For this purpose, fMRI and EEG measurements were performed in a counterbalanced order. In the third and fourths sessions, TMS was used to perturb two regions of interest (one in each TMS session) which were obtained from group peak activations of the fMRI session. Data of the EEG measurements will be presented elsewhere.

**Figure 1:**
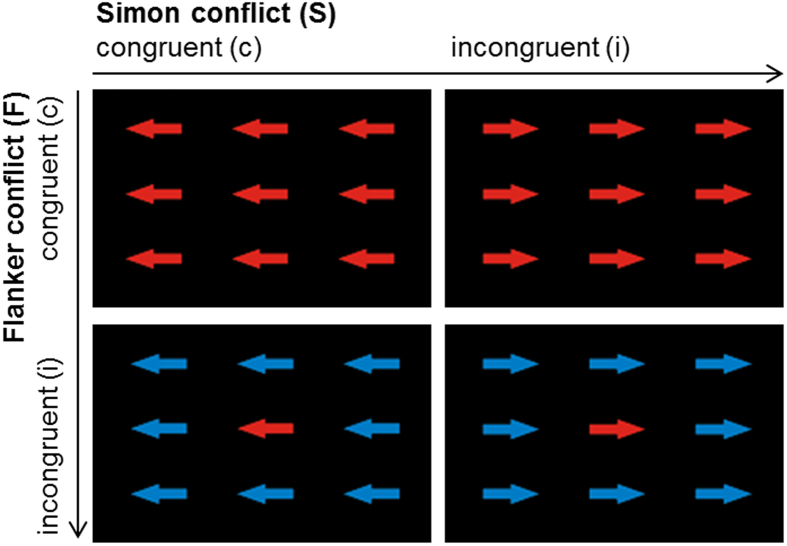
Example for the respective four experimental conditions. Subjects were asked to respond to the color of the target by either pressing a button with the left or right hand (in this case subjects were instructed to press with the left hand if the colour of the central error was red and with the right hand if the colour was blue). The target stimulus was flanked by eight distractor arrows which either had the same color (congruent Flanker (S-S) condition, Fc; upper left) or the alternative color (incongruent Flanker condition, Fi; lower left). The direction into which the arrows were pointing was either the same as the expected response side (congruent Simon (S-R) condition, Sc, upper left) or the opposite side (incongruent Simon condition, Si, upper right). The lower right panel shows the double conflict condition (FiSi) with incongruent Flanker and Simon conditions.

Subjects performed the same experimental task in all sessions. Stimuli were presented on a black background. Each trial started with a white fixation cross presented for 800 ± 150 ms. Subsequently, the target display was presented for 250 ms, followed by a black screen for 2200 ms until the next trial started. The target display consisted of nine blue or red arrows (see Fig. 1). The central arrow was the target stimulus and subjects were asked to respond to the color of the target by either pressing a button with the left or right hand. Half of the subjects were asked to press left if the central arrow was red and right if central arrow was blue, the other half of the subjects had to perform the opposite mapping. The target stimulus was flanked by eight distractor arrows which either had the same color (congruent Flanker (S-S) condition, Fc) or the alternative color (incongruent Flanker condition, Fi). All arrows pointed either to the right or left. Thus, the direction into which the arrows were pointing was either the same as the expected response side (congruent Simon (S-R) condition, Sc) or the opposite side (incongruent Simon condition, Si). Consequently, there were four different conditions in the experiment (see Fig. 1): (i) Flanker and Simon congruent (FcSc), (ii) Flanker incongruent, Simon congruent (FiSc), (iii) Flanker congruent, Simon incongruent (FcSi), and (iv) both Flanker and Simon incongruent (double conflict, FiSi). Each condition consisted of the same number of trials. Note that even though all conditions had comparable visual and motor input with coloured arrows pointing to certain directions, requiring a manual response, the four conditions varied with respect to the presence of a Flanker or Simon conflict. Condition (i) had neither a Flanker nor a Simon conflict, since the colour of all stimuli was the same and the direction was the same pointing to the side of the response hand. In condition (ii) the direction of the central arrow pointing to the side of the response hand but the colour of the central arrow was different from the surrounding arrows leading to a Flanker conflict only. In condition (iii) the colour of all stimuli was the same but the direction of the central arrow pointed to the side opposite of the response hand inducing a Simon conflict only. Condition (iv) combined both conflicts with the central arrow pointing to the side opposite of the response hand. In addition, the colour of the central arrow was different from the surrounding arrows.

Participants were asked to respond as quickly and accurately as possible by pressing a button on a mouse device placed in their left and right hand, respectively. The mapping between stimulus color and response side was pseudo-randomly distributed across all subjects. The task consisted of 576 trials for the fMRI session and 288 trials for the TMS sessions. Stimuli were presented in blocks of 48 trials which were separated by a break lasting 10 seconds. Trial order was pseudo-randomized and different for each of the participant’s runs. Within each run the sequence of congruent and incongruent trials was counterbalanced to prevent sequence-effects. Stimuli were presented with the Presentation software (Neurobehavioral Systems, http://www.neurobs.com, Berkeley, USA).

### Analysis of behavioral data

Mean reaction time (RT) of all correct trials and error rate (ER) was computed for each participant, condition and session separately. For the analysis of the behavioral data of both the fMRI and TMS sessions, repeated measures analyses of variance (ANOVA) with the within-subject factors flanker congruency (congruent/incongruent) and Simon congruency (congruent/incongruent) was computed separately for error rate and RT. For the TMS component, a third factor TMS condition (active TMS/Sham TMS) was included.

### fMRI data acquisition and analysis

Scanning was performed on a 3-T SIEMENS Magnetom Allegra^®^ system (Siemens, Erlangen, Germany). Subjects wore foam earplugs and were positioned on a scanner couch in a dimly lit room. Stimuli were presented via a projector onto a projection screen, visible to the subject via a mirror attached to the head coil. A T2*-weighted echo-planar imaging sequence (TR = 1500, TE = 30 ms, flip angle = 73°, 28 contiguous axial slices aligned to the AC-PC line, slice thickness = 4 mm, no gap, 64 × 64 matrix, in-plane resolution = 3 × 3 mm, interleaved acquisition order) was used to obtain the fMRI data. Additionally, a T1-weighted structural 3D-image of the brain was obtained using a MPRAGE sequence (TR = 2.3s, TE = 4.38 ms, flip angle = 8°, TI = 900 ms, 176 contiguous slices, FOV = 256 × 256 mm, in-plane resolution = 1 × 1 mm, slice thickness = 1 mm). The processing and analysis of imaging data was performed with SPM8 software (Wellcome Department of Cognitive Neurology, London, UK; see http://www.fil.ion.ucl.ac.uk/spm). The first 5 volumes were discarded to allow for T1 equilibration effects. Scans were re-aligned to the first volume, slice-time corrected and spatially normalized to the standard SPM EPI template in MNI space. Smoothing was conducted by use of a three-dimensional Gaussian filter of 8 mm full-width-at-half-maximum to compensate for interindividual anatomical variability. A high-pass filter (128 Hz) and a correction for temporal autocorrelation in the data (AR 1 + white noise) were applied to accommodate serial correlations.

Statistical analyses of the functional imaging data were performed in a two-level mixed effects analysis. At first level, four regressors of interest representing the four conditions were modeled for each subject using a standard hemodynamic response function with temporal derivatives. Additional regressors were included for erroneous trials as well as six motion regressors containing movement parameters obtained during realignment. Weighted contrasts coding for the fMRI signal increase in the four conditions of interest were entered into a second level ANOVA. On the second level we analysed the main effect of Flanker conflict and Simon conflict using a pooled error model and the appropriate F-contrasts^47^. Additionally, an a priori contrast testing for higher neural activity in the combined Flanker and Simon conflict as compared to trials with either only Flanker or Simon conflict (FiSi-(FiSc+FcSi)) was performed. In other words, we compared the condition with a double conflict to the two conditions encompassing the respective simple conflict to isolate brain regions that contribute to double as compared to single conflict. Significant activations are reported at p < 0.05 corrected for multiple comparisons on cluster level for *a priori* t contrasts or voxel level (family-wise-error, FWE, cluster extent threshold of k ≥ 20) for F contrasts. The statistical parameters were chosen in a way to find a small number of selectively activated areas for each conflict type as a prerequisite for the TMS experiment. Coordinates correspond to the standard Montreal Neurological Institute (MNI) brain.

### TMS data acquisition

All participants were tested in two experimental sessions, performed on two separate days, involving repetitive TMS (rTMS) of two different right hemispheric regions. In each experimental session, the experimental task (consisting of six blocks with 48 trials each) was run three times, at baseline (without TMS), after sham stimulation and after active rTMS. The target regions for rTMS were derived from the fMRI group data (see below). As a first region we chose the right IPS since this area showed strongest fMRI activation for the flanker interference. Second, the right MFG was selected, because this region showed increased fMRI activation for the double conflict compared to the single conflict conditions. Both stimulation sites were determined by using the high-resolution structural 3D-image of each subject in conjunction with the BrainSight frameless stereotaxy system (BrainSight Frameless, Rogue Research, Montreal, Canada). The respective fMRI group activation was converted into each subject’s individual brain anatomy and plotted onto the structural image. Cortical target locations were than projected onto corresponding points of the subjects scull and scull locations were tracked by using the BrainSight frameless stereotaxy system. Stimulation of the respective target regions was counterbalanced. Half of the participants were first tested with rTMS over IPS, the other half over MFG.

The rTMS was delivered via a 70 mm figure-of-eight coil, connected to a Magstim Super Rapid stimulator (Magstim, Whitland, UK). Stimulation consisted of 20 minutes 1 Hz rTMS, at 65% intensity of the stimulator. During active stimulation, the center of the coil was placed over the stimulation site such that the two rings extended in the anterior and posterior directions with the handle pointing away from the cortex, perpendicular to the mid-line. Sham stimulation was performed with the coil positioned vertically to the scalp surface, with the lateral, narrow edge of the rings touching the scalp at the stimulation site^48^. This rTMS paradigm was expected to lead to diminished activity at the stimulation site following the stimulation^49^.

## Results

### Behavioral data

Error rates and reaction times for the four experimental conditions are shown in Table 1. For the fMRI session, the 2 × 2 ANOVA showed significant main effects of Flanker (error rates: *F*(1,16) = 8.4, *p* = 0.01; RT: *F*(1,16) = 155.8, *p* < 0.001) and Simon congruency (error rates: *F*(1,16) = 6.7, *p* < 0.05; RT: *F*(1,16) = 17.1, *p* = 0.001). The interaction of Flanker x Simon congruency was not significant (error rates: F(1,16) = 1.5, p > 0.05; RT: F(1,16) = 0.2, p > 0.05).

For the baseline data analysis of the TMS experiment we combined and jointly analyzed the baselines for the IPS and the MFG since there were no significant differences between both sessions. The 2 × 2 ANOVA revealed a significant main effect of Flanker congruency (error rates: *F*(1,14) = 9.3, *p* < 0.01; RT: *F*(1,14) = 67.8, *p*  < 0.001) and Simon congruency (error rates: *F*(1,14) = 8.5, *p* < 0.05; RT: *F*(1,14) = 15.8, *p* = 0.001). There was no significant interaction of Flanker x Simon congruency (error rates: (*F*(1,14) = 0.1, *p* > 0.05; RT: *F*(1,14) = 0.1, *p* > 0.05).

When comparing the fMRI and TMS baseline data, the analysis for error rates did not show significant differences (*F*(1,14) = 1.9, *p* > 0.05). However, RTs were significantly slower in the fMRI compared to the TMS session (*F*(1,14) = 19.2, *p* = 0.001) and the Flanker effect was significantly stronger in the fMRI than TMS session (*F*(1,14) = 8.5, *p* < 0.05). These differences in RTs are either attributable to session effects or the noisy, more uncomfortable and more distracting environment in the fMRI scanner.

### Functional imaging data

**Figure 2:**
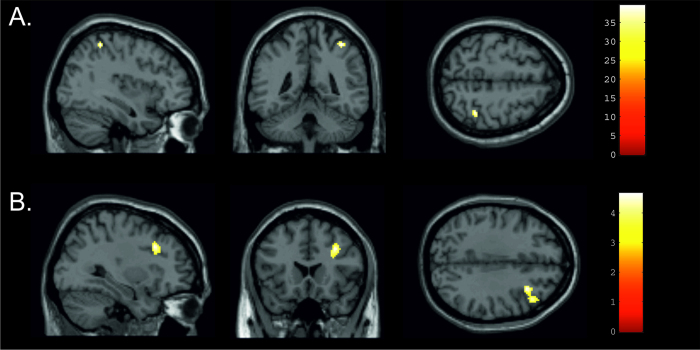
Regions involved in conflict processing. (**A**) Neural activity related to the Flanker conflict. The right intraparietal sulcus (IPS) shows significantly higher neural activity in trials with incongruent (Fi) compared to congruent (Fc) Flanker stimuli (FiSc+FiSi vs. FcSc+FcSi). Activations are displayed on an individual structural MRI at p < 0.05 corr. at voxel level. (**B**) Neural activity related to the combined Flanker and Simon conflict. The right middle frontal gyrus (MFG) shows significantly higher neural activity in trials with both, incongruent Flanker and Simon stimuli (FiSi > FiSc+FcSi). Activations are displayed on an individual structural MRI at p < 0.05 corr. at cluster level. The colour bars indicate the Z-score associated with each voxel.

The Flanker effect (FiSc+FiSi vs. FcSc+FcSi) was associated with a significant increase in BOLD activity in the right intraparietal sulcus (IPS; peak MNI coordinate: [38 −44 56], Z = 5.52, p(corr) = 0.002, k = 24; see Fig. 2A) with higher neural activity for incongruent than congruent flanker stimuli. No significant increases in BOLD activity were found for the Simon effect (FcSi+FiSi vs. FcSc+FiSc) or the Flanker by Simon effect interaction. The combined Flanker and Simon effect (FiSi > FiSc+FcSi) revealed significant BOLD signal changes in the right middle frontal gyrus (MFG; peak MNI coordinate: [32 20 34], Z = 4.3, p(corr) = 0.023, k = 236; see Fig. 2B) with significantly higher signal increases in trials where conflict was present in both dimensions.

### TMS data

Based on the data presented above, TMS pulses were delivered over the activation maxima in the right IPS and MFG to test whether perturbations affect the Flanker effect and the combined Flanker and Simon effect, respectively. Behavioral effects were analyzed by conducting 2 × 2 × 2 repeated measures ANOVAs with the factors TMS condition, flanker congruency and Simon congruency for error rates and RTs separately.

#### Stimulation of the IPS

**Figure 3:**
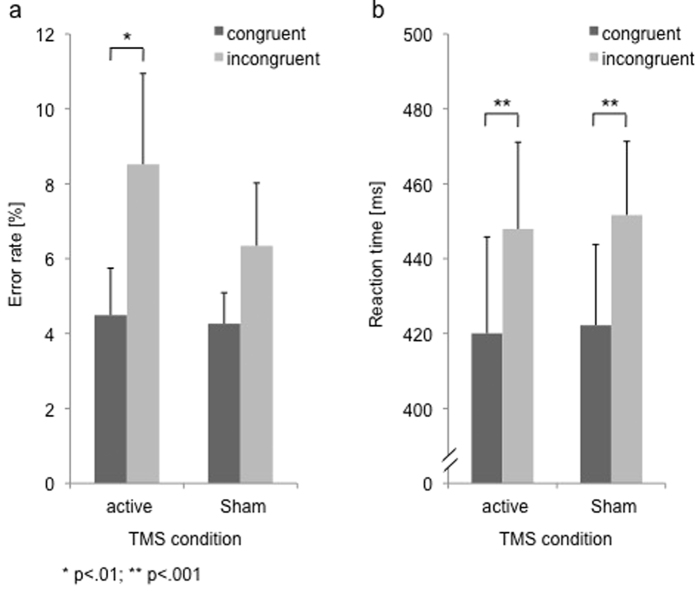
(**a**) Errors rates (in %) and (**b**) reaction times (in ms) for the active and Sham TMS of the right IPS for the congruent and incongruent flanker condition (pooled over both Simon conditions). Error bars represent standard errors of the mean.

Figure 3 depicts error rates and reaction times after active and sham stimulation of the IPS. The ANOVA for the error rates resulted in significant main effects of Flanker congruency (*F*(1,14) = 7.7, *p* < 0.05) and Simon congruency (*F*(1,14) = 8.8, *p* = 0.01). Subjects responded more accurately for congruent (4.4% errors) than incongruent Flanker stimuli (7.4%) and for congruent (4.3%) than incongruent Simon stimuli (7.5%). There was no significant main effect of TMS condition (*F*(1,14) = 3.4, *p* > 0.05). We found, however, a significant interaction of TMS condition x flanker congruency (*F*(1,14) = 6.9, *p* < 0.05). This interaction resulted from a significant Flanker effect after active TMS (t(14) = 3.1, p < 0.01), but a non-significant effect after Sham TMS (t(14) = 2.0, p > 0.05; see Fig. 3). No other interactions reached significance (all F < 1.8, all p > 0.05). Thus, TMS affected the single conflict condition of the Flanker task, but none of the other conflict conditions.

For the RTs, the analysis revealed a significant main effect of Flanker congruency (*F*(1,14) = 77.0, *p* < 0.001) and Simon congruency (*F*(1,14) = 13.0, *p* < 0.01). Responses were faster for congruent (421 ms) than incongruent flanker stimuli (450 ms) and for congruent (429 ms) than incongruent Simon stimuli (442 ms). There was no main effect of TMS-condition (*F*(1,14) = 0.2, *p* > 0.05) and no significant interactions (all F < 0.8, all p > 0.05).

#### Stimulation of the MFG

The second region chosen for TMS perturbation was the right MFG. Analyzing the effects on error rates, the ANOVA showed significant main effects of Flanker congruency (*F*(1,14) = 7.5, *p* < 0.05) and Simon congruency (*F*(1,14) = 6.8, *p* < 0.05). Subjects made fewer errors for congruent (4.0% errors) than incongruent Flanker stimuli (6.8%) and for congruent (4.2%) than incongruent Simon stimuli (6.5%). There was no significant main effect of TMS condition (*F*(1,14) = 1.0, *p* > 0.05) and no significant interactions (all F < 0.6, all p > 0.05).

For the RTs, the ANOVA showed a strong main effect of Flanker congruency (*F*(1,14) = 165.7, *p* < 0.001). Subjects responded faster for congruent (433 ms) than incongruent Flanker stimuli (462 ms). There was no significant main effect of Simon congruency (*F*(1,14) = 4.2, *p* > 0.05) and TMS condition (*F*(1,14) = 1.3, *p* > 0.05). Likewise the analysis revealed no significant interactions (all F < 1.1, all p > 0.05).

## Discussion

In this fMRI/TMS study we investigated whether different conflict types are processed separately in different brain regions and whether they are differentially vulnerable to TMS perturbation. We used a combined S-S (Flanker) and S-R (Simon-like) task and identified the brain regions involved in processing the interference induced by the different single conflicts and the combined double conflict. Potential causal functional contributions to these conflict types were then examined by TMS perturbation of selected activated brain areas.

As expected, the analysis of the behavioral data revealed significant interference effects. Regarding the Flanker conflict, participants showed a Flanker effect with significantly more errors and significantly longer RTs for incongruent than congruent trials. Similarly, the Simon conflict led to significantly more errors and significantly longer RTs in response side incongruent compared to congruent trials. At the behavioral level, there was no significant interaction between the Flanker and Simon interference.

### FMRI data

The fMRI data yielded different activation maxima for the flanker and double conflict condition. The Flanker conflict yielded highest activations in the right IPS, a region that was not activated by the Simon or double interference condition. The parietal cortex around the IPS is assumed to be involved in conflict resolution by orienting attention to the relevant target^17^,^20^,^21^, and additionally in the representation of possible responses^22^,^23^. In particular, the orienting of attention is important for the Flanker but not Simon conflict. In the Flanker task, attention has to be oriented to the target and flankers have to be ignored. In the Simon task, it is the spatial dimension of the target that produces the conflict. Thus, the conflict is assumed to be resolved at the response level that should be processed in frontal brain regions.

For the Simon conflict, we did not find any selectively activated brain region that survived correction for multiple comparisons. The behavioural data also show that the interference induced by the S-R (Simon) conflict is less strong than for the S-S (Flanker) conflict implying that brain activation is on a lower level and potentially less widespread. This finding is in line with the data of previous studies of our group^7^,^11^ that also showed only sparse or no activation for the S-R conflict condition.

In contrast to the single conflicts, the double conflict activated the right MFG. This region is part of the DLPFC that is assumed to monitor conflicting information^17^,^20^,^21^,^24^, and to be involved in response selection^18^,^22^,^25^,^26^. Activity in the right MFG also increases with increasing distraction and attentional effort^50^. All of these processes are relevant for the simultaneous processing of Flanker and Simon conflicts. Especially in the double conflict condition, there is an increased demand on monitoring interfering information to resolve both conflicts. Also, the demands on selection among competing responses are higher in the double conflict compared to the single conflicts.

The fMRI data of the current study support the findings of our prior study^7^ that demonstrated different conflicts to activate distinct rather than common brain areas. To further examine the selectivity of activation due to different conflicts, these selectively activated brain regions were used to examine the differential vulnerability of the conflict types to TMS.

### TMS of the right IPS

As discussed before, the Flanker conflict selectively activated the right IPS. We did not find activation in that region for the Simon or double conflict. As therefore expected, TMS of the right IPS only affected the Flanker conflict but not the Simon or double conflict condition. For error rates, the Flanker effect increased after TMS compared to the Sham condition. In a previous study using unilateral flankers, TMS of the right PPC in the vicinity of the IPS (10–20 coordinate point P4) reduced the Flanker effect for flankers in the contralateral left visual field^38^ while the flanker effect for ipsilateral flankers was not affected. This data might be explained by reduced spatial attention to flankers in the left visual field after TMS of the right IPS inducing a decreased flanker effect. This means that in a Flanker task with unilateral flankers, TMS disturbed spatial attention. In the current study, however, we did not use unilateral but bilateral flanker stimuli. Furthermore, we found an increase of Flanker interference after TMS. Thus, reduced spatial attention for the left visual field probably does not account for the TMS effect in the present study. Soutschek *et al*.^39^ also found an increase of Flanker induced interference after the stimulation of the IPS. The authors argued that the stimulation possibly interferes with attentional top-down mechanisms, thereby impeding the identification of the relevant stimulus information. Consistently, several fMRI studies suggest that the parietal cortex is associated with attentional biasing of relevant and irrelevant information during the resolution of conflict. In the context of a Flanker task, reduction of interference can be achieved by narrowing the attentional focus to the target stimulus, thereby reducing the impact of the incongruent distracters. Thus, the activation of the IPS in the present study possibly indicates the modulation of the attentional focus in incongruent Flanker trials. Perturbation of the IPS possibly disrupts this mechanism and leads to an amplified processing of the distracters associated with an increased Flanker effect (as indicated by increased error rates).

In contrast, Soutschek *et al*.^39^ did not find any effect of IPS stimulation on the Simon conflict. This finding is in line with previous studies that also reported independent neural networks to be in charge for the processing of S-S and S-R conflict types. Our data also corroborate the finding that the IPS seems to be critically involved in the processing of S-S conflict types while activation/perturbation of the IPS is not necessarily associated with the processing of S-R conflicts. Consistent with previous fMRI data, we also found no significant change of behavioral measures in double conflict trials after TMS of the IPS. These data indicate that the cognitive mechanisms associated with the IPS do not provide an effective way to reduce or resolve interference in the context of S-R conflicts. Since relevant and irrelevant information in the Simon task are confounded in the same stimulus setting, the narrowing of the attentional focus does not decrease the impact of the distracting stimulus characteristics as in the Flanker task. Thus, other cognitive mechanisms such as the modulation of motor-related control processes are possibly involved in the resolution of S-R conflicts. Likewise, in double conflict trials, the modulation of the attentional focus possibly plays a minor role compared to single Flanker conflict trials, because this mechanism seems not sufficient to resolve both sources of conflict. Thus, the activation of the IPS in double conflict trials is probably less consistent and not associated with TMS induced perturbation and behavioral performance.

Considering the debate of the interdependency of S-S and S-R conflicts, our findings rather suggest that both conflict types are linked to distinct neural networks. The TMS data of the IPL perturbation also suggests that the mechanisms involved in the resolution of a specific conflict are adaptive with regard to demands of a specific situation, for instance, the co-occurrence of another conflict.

### TMS of the right MFG

For the double conflict condition we found an activation of the right MFG that was not found for the single conflicts. As mentioned above, the activation might be a result of a specific involvement of MFG in processing combined S-S and S-R conflicts, or more generally due to an increased cognitive demand in a double conflict condition to monitor conflicting information^17^,^20^,^21^,^24^ and to select a response^18^,^22^,^25^,^26^. However, TMS of the right MFG had neither an effect on the double conflict condition nor on the single conflict conditions. Thus, the present findings do not allow us to assign a causal functional role in conflict processing to MFG.

There are several possible reasons for why we did not find a TMS effect for the double conflict. It might be that the double conflict is too strong and robust to be disturbed by TMS, or that the activation observed in the MFG is a redundant epiphenomenon that is not causally underlying the processing of double conflicts. However, it is also possible that, due to technical factors, the right MFG was not easily accessible via TMS. The activation pattern varied between participants and not all of them showed activation of the right MFG for the double conflict condition, even if the group analysis indicated a strong activation. Thus, it might be that not for all participants the stimulation involved the relevant area. In addition, the group activation in the right MFG was rather small and appeared to be located particularly deep in the brain (cf. Fig. 2). Thus, it could be that it was not possible to reach the relevant brain region with TMS.

## Conclusion

In summary, our study demonstrated that different conflict types activate distinct brain areas and can be selectively perturbed by TMS. In particular, TMS perturbation of the intraparietal sulcus selectively affected the processing of the Flanker (S-S) conflict. These findings underscore the idea that different conflict types are processed independently. For future studies, it will be worthwhile to make a further effort to address the specific causal functional role of the MFG and other brain regions in processing combined, multiple conflict conditions.

## Additional Information

**How to cite this article:** Peschke, C. *et al*. Selective perturbation of cognitive conflict in the human brain–A combined fMRI and rTMS study. *Sci. Rep.* **6**, 38700; doi: 10.1038/srep38700 (2016).

**Publisher's note:** Springer Nature remains neutral with regard to jurisdictional claims in published maps and institutional affiliations.

## Figures and Tables

**Table 1:** Errors rates (in %) and reaction times (in ms) for the fMRI and TMS baseline session.

	Experimental conditions			
	FcSc	FiSc	FcSi	FiSi
*fMRI*				
Error rates	2.3 (0.4)	5.2 (1.5)	4.0 (1.0)	8.2 (1.9)
RT	501 (18)	538 (18)	520 (19)	554 (18)
*TMS baseline*				
Errors rates	2.6 (0.5)	6.3 (1.5)	5.1 (1.1)	9.2 (2.4)
RT	435 (27)	463 (24)	447 (27)	476 (25)

Numbers in brackets represent standard errors of the mean. FcSc–congruent condition: no conflict, FiSc–Flanker (S-S) conflict, FcSi–Simon (S-R) conflict, FiSi–double conflict.
